# The Dark Side of Boys’ Compliments to Girls: Exploring Their Relationship with Sexism and Cyberviolence Towards Intimate Partners

**DOI:** 10.3390/bs15050572

**Published:** 2025-04-24

**Authors:** Yolanda Rodríguez-Castro, Rosana Martínez-Román, María Lameiras-Fernández

**Affiliations:** Faculty of Education and Social Work, University of Vigo, 32004 Ourense, Spain; rosana.mr@uvigo.gal (R.M.-R.); lameiras@uvigo.gal (M.L.-F.)

**Keywords:** compliment, sexism, cyberviolence, partners, adolescent

## Abstract

The objective of this study was to evaluate the frequency with which boys “compliment” girls, know their perceptions about whether girls like compliments, and discen whether they believe that society expects them to make such comments. The relationship of such compliments with the level of ambivalent sexism and cyberviolence towards the partner was also evaluated. A total of 498 adolescent boys participated in this study, with a mean age of 16.01 years (SD = 1.02), recruited with the Computer-Assisted Web Interviewing (CAWI) system. The main results obtained show that younger boys more frequently emit objectifying messages about women’s bodies than older boys. They believe these comments positively impact girls, thinking they are appreciated. These boys, especially younger boys, show higher levels of hostile and benevolent sexism and perform more cyberviolence towards their partners. Boys’ level of partner cybercontrol predicts the emission of comments about women’s bodies, especially in boys with a high level of hostile sexism. Therefore, to prevent sexual harassment, gender-based cyberviolence, and sexism, it is crucial for the educational system to promote comprehensive sex education.

## 1. Introduction

The issue of the objectification of women’s bodies has generated a proliferation of research (Lameiras et al., 2018; Manago et al., 2015; Moradi & Huang, 2008; Pecini et al., 2022), especially as a result of the development of the theory of objectification of women (Fredrickson & Roberts, 1997). The theory of objectification developed a “schema for understanding the consequences of being a woman in a culture that sexually objectifies women’s bodies” (p. 173). Objectification messages also activate experiences of self-objectification, placing women in the perspective of an observer or third person, making them efficient observers of their own body and its main controller (Fredrickson & Roberts, 1997). According to the objectification theory, women’s identity has been reduced to their beauty and sexuality, so they link their identity to their bodies and are doomed to dissatisfaction when they do not submit to beauty standards. These experiences of objectification have serious consequences for women’s health and well-being (Manago et al., 2015; Niu et al., 2020; Tiggemann & Slater, 2015).

### 1.1. Objectification of Women Through “Compliments”

According to Fredrickson and Roberts (1997), forms of sexual objectification are expressed through body evaluation (comments about the body) and unwanted sexual advances, which are less frequent (Kozee et al., 2007). Thus, the experiences of objectification that women receive in interpersonal relationships materialize in behaviors such as the visual inspection of their body or body parts, whistling, sexual comments about the body or body parts that are neither authorized nor reciprocated by the women, deriving in unwanted sexual advances, sexual harassment, and abuse. Therefore, comments about the sexual body materialized in the form of “compliments” represent the most frequent experiences (Vera-Gray, 2016), still insufficiently studied. Janet Holmes (1988) defines the compliment as “a linguistic act that explicitly or implicitly attributes credit to someone other than the sender, usually the person to whom the comment is addressed, for something “good” (a possession, characteristic, ability, etc.), which is valued by the sender and the receiver” (p. 446). Although not included in its definition, Holmes (1988) states that compliments represent messages focused mostly on women’s appearance and bodies, as opposed to the compliments received by men, which are mainly focused on their abilities.

The compliment represents the “reinforcement” of a way of being (descriptive stereotypes) and behaving (prescriptive stereotypes), based on the attributes considered specific to each gender (Rodríguez-Castro et al., 2012). Compliments are mandated by gender stereotypes: the “gallantry” of men (as senders of compliments) versus the “beauty” of women (as recipients of compliments) (Lameiras et al., 2018). In the practice of complimenting, men conform to prescriptive stereotypes of masculinity (Salter, 2016), and it helps them to reinforce their image both to themselves and to society. Thus, compliments can represent a mark of the “territory” under the domination of men, who turn compliments into messages of threat or deterrence toward women who dare to walk through a public space under male domination and control (Lameiras et al., 2021).

This type of comment that sexually objectifies a woman’s body or body parts through compliments has become a form of sexual harassment (Moya-Garófano et al., 2020). Compliments are the most common and normalized sexual harassment in Western cultures in countries such as Spain, Portugal, and other Spanish-speaking countries (Bailey et al., 2016; Moya-Garófano et al., 2022), which could lead to these behaviors being socially considered positive by society and/or by the recipient (Moya-Garófano et al., 2020, 2021).

Experiences of objectification, especially through comments about women’s bodies or compliments, often begin in the transition from childhood to adolescence, when girls’ bodies begin to attract the gaze, sexualization, and evaluation of boys and men (Fredrickson & Roberts, 1997; Rousseau & Eggermont, 2017; Tiggemann & Slater, 2015; Ward et al., 2015). During adolescence, physical appearance and physical attractiveness confer a social status of acceptance (e.g., if they are handsome or sexy) or rejection (e.g., if they are ugly or do not meet the standard of a lean, athletic body, etc.) within the peer group, especially affecting girls (Mayeux & Kleiser, 2020). Thus, the sexual objectification of the body becomes a problem in the lives of adolescent girls, extending into adulthood (Fredrickson & Roberts, 1997).

The level of women’s sexual objectification may be conditioned by the influence of the type of content that adolescents view in the mass media. In fact, the boys who most visualize mass media objectifying women’s bodies (e.g., music, sexualized magazines, pornographic websites, etc.) are more likely to perceive girls/women as sex objects (Ward et al., 2015; Wright & Tokunaga, 2016) and may even show a higher level of sexual violence (Wright & Tokunaga, 2016). As evidenced in the study by Ward et al. (2015), exposure to sexualized mass media has a direct impact on how adolescents communicate and relate to each other, especially on the strategies that boys deploy to “flirt” with girls, as they may mistakenly perceive the girl’s physical appearance as a sexual invitation, triggering some behavior of sexual violence. The following reasons are given by heterosexual boys and men aged 16 to 75 who make objectifying comments to women (Walton & Pedersen, 2022): sexual attractiveness (72%), to show that they like them (85.4%), to show sexual interest in women (82.9%), to flirt (73.1%), and because they think that women like to receive this type of comment (74.4%). After making sexual comments, 54.9% of the study participants expect the woman to agree to a date or to have sex with them (52.4%).

### 1.2. Ambivalent Sexism and Objectification Through Compliments

Ambivalent sexism is a two-dimensional construct comprising hostile and benevolent attitudes toward women (Glick & Fiske, 1996, 2001). Both types of sexism function as complementary ideologies and as a reward and punishment system: Hostile sexism, with a negative tone, considers women inferior to men. It is applied as a punishment to women who do not fulfill the traditional roles of wife, mother, and caregiver. In contrast, benevolent sexism, with a positive affective tone, considers women to be different and, as such, it is necessary to care for and protect them, so traditional women are rewarded with benevolent sexism (Rodríguez-Castro et al., 2021). Adolescents have ambivalent sexist attitudes, with boys being more sexist, hostile, and benevolent than girls (Gutiérrez et al., 2020; Navas et al., 2020).

Several studies have shown that ambivalent sexism is related to the importance of physical appearance for women and to experiences of sexual objectification (Moya-Garófano et al., 2022; Wright & Tokunaga, 2016). Hostile sexism correlates positively with the importance men grant to women’s beauty, thinness, and physical attractiveness (Forbes et al., 2007; Lee et al., 2010). Benevolent sexism influences women to reproduce the prescribed gender stereotypes and roles (Glick & Fiske, 2001). According to these sexist attitudes, women may even experience an increase in their self-esteem or feel empowered when they attract sexual attention from men through sexual comments about their physical appearance (Liss et al., 2011).

The study by Harsey and Zurbriggen (2021) shows that adolescents’ acceptance of objectification towards women is associated with sexist attitudes. Boys show a greater acceptance of objectification towards women’s bodies than girls, and they also show higher levels of hostile and benevolent sexism than girls. In the Spanish context, the study by Moya-Garófano et al. (2022) shows that women with a higher level of hostile sexism have positive attitudes towards compliments and experience happiness, feelings of power, and less anger, hostility, and anxiety. However, women with a high level of benevolent sexism do not show positive attitudes toward compliments.

### 1.3. Cyberviolence in Relationships and Compliments

In the online space, young people have found a new way to reproduce and perpetuate the objectification of women’s bodies (Burnell et al., 2021; Lin et al., 2022; Tiggemann & Barbato, 2018), sexism (Drakett et al., 2018), and cyberviolence in relationships (Cava et al., 2020; Rodríguez-Castro et al., 2021).

Currently, according to the study by Burnell et al. (2021), 60% of adolescents within relationships exchange photos, videos, or comments focused on the physical or sexual attractiveness of their partner. In the case of boys, their comments allude to specific parts of their partner’s body, such as the buttocks and breasts, because this excites them. This situation can lead to cyberviolence against the partner. Intimate partner cyberstalking (IPCS) is defined as “cyberstalking behavior towards current, former, or potential intimate partners” (Smoker & March, 2017, p. 391). Studies focusing on IPCS in adolescents indicate that the most common behavior is usually the online control of one’s partner (Reed et al., 2017; Van Ouytsel et al., 2019). Within the range of partner cybercontrol behaviors in adolescents, the following stand out: sending insulting and/or threatening messages to the partner and/or controlling where and with whom the partner is by means of instant messaging applications or social networks (Calvete et al., 2021).

In this sense, the study of Rodríguez-Castro et al. (2021) shows that boys and girls admit to controlling their partner in the virtual space. However, adolescent girls are more cybercontrolling of their partner because they consider controlling behavior as a way to express love and care for a partner and as an “effective” tool to maintain their partner relationship (Smoker & March, 2017; Rodríguez-Castro et al., 2021).

### 1.4. The Present Study

The study on the experiences of sexual objectification through compliments is still a developing line of research in the Spanish context, focused on the frequency or impact of sexual comments received by university or adult women from strangers. We highlight the study of Moya-Garófano et al. (2016), in which 25.9% of young women admitted to suffering sexual harassment in the street through compliments, or the study of Ferrer-Pérez et al. (2021), in which 94.7% of girls had received compliments in the street at least once. In addition, most women reject these types of comments about their bodies (Moya-Garófano et al., 2022). It has been shown that compliments are harmful in the short or medium term for women, regardless of whether they experience them positively or negatively (Moya-Garófano et al., 2021).

Intending to take a step further in the study of objectification through comments about women’s bodies, this research focuses on adolescent boys as the main emitters of this type of comments to determine the frequency with which they emit “compliments” to girls, to know their perceptions and beliefs about whether girls like these types of comments as well as the positive or negative effect it can have on them. This research also examines whether boys believe that society expects them to make such comments and whether they would be willing to stop issuing them if a girl or woman expressed their disagreement with them. We also intend to assess the level of ambivalent sexism and cyberviolence performed towards their partner and to verify its relationship with the emission of “compliments” or comments about women’s bodies.

## 2. Method

### 2.1. Participants

The participants were 498 Spanish adolescent boys, ranging from 14 to 18 years, with a mean age of 16.01 (SD = 1.02), who consented to complete an online anonymous survey to achieve the study’s objectives. In this study, two selection criteria were considered: being a heterosexual boy and currently having a partner or having had a partner in the past for at least 6 months. Of the total sample, 89.8% (n = 447) of the boys defined their sexual orientation as heterosexual (3.5% as bisexual and 3.7% as homosexual), and of them, 70.69% had or had had a partner. Thus, the sample of this study was made up of 316 heterosexual adolescent boys.

### 2.2. Measures

For this study, we used a questionnaire comprising the following items and scales:

*Demographic questions*: The male participants indicated their age and sexual orientation. Responses ranged from 1 (fully heterosexual) to 5 (fully homosexual).

*Relational History Issues*: They were asked if they had been in a relationship for at least 6 months and if they currently had a partner.

*Frequency with which comments about the body are emitted*: They were asked how often they make comments about women’s bodies (ad hoc): Responses were rated on a Likert scale ranging from 1 (never) to 5 (very often).

*An evaluation of the impact they consider that comments about the body have on women*: They were asked the following question (ad hoc): do you think girls like to receive comments about their bodies? Responses were rated on a Likert scale ranging from 1 (strongly disagree) to 6 (strongly agree).

*Boys’ perception of the effect of comments on women’s bodies*: Five items were included to know boys’ perception of the effect that comments about the body have on women. The items begin with the stem phrase (ad hoc) “Do you think that when women receive compliments or comments about their bodies, they find it…?”, with five options: funny, pleasant, gallant, offensive, and annoying. Responses were rated on a Likert scale ranging from 1 (strongly disagree) to 6 (strongly agree).

*Identify societal expectations about the practice of commenting on women’s bodies*: To determine whether the participants think they are expected to comment on women’s physical appearance, they were asked the following question (ad hoc): “Do you think you’re expected to give compliments and/or comments about women’s physical appearance?” Responses were rated on a Likert scale ranging from 1 (strongly disagree) to 6 (strongly agree).

*Predisposition to change*: To determine whether boys would stop commenting on women’s bodies if women informed them of their discomfort, the following question was asked (ad hoc): “If a woman about whose body you make comments were to respond to you informing you of the discomfort it causes her, would you stop doing it?” Responses were rated on a Likert scale ranging from 1 (strongly disagree) to 6 (strongly agree).

*Ambivalent Sexism Inventory* (Glick & Fiske, 1996): This study used the short 12-item version of the Ambivalent Sexism Inventory (the Spanish version by Rodríguez-Castro et al., 2009). This scale consists of 12 items: 6 items measure hostile sexism (the most traditional sexism, which considers women inferior to men) and the remaining 6 items measure benevolent sexism (which, with a positive affective tone, considers women not inferior to men but different). Responses are rated on a 6-point Likert scale ranging from 0 (strongly disagree) to 5 (strongly agree). Higher scores indicate higher levels of hostile sexism and benevolent sexism. In our study, Cronbach’s alphas were 0.85 and 0.83, respectively, for the hostile sexism and benevolent sexism subscales.

*Cyberdating Scale Q-A* (Sánchez et al., 2015): This scale assesses cyberviolence in adolescent relationships through 28 items organized into six subscales that measure online intimacy, emotional communication strategies, cyber practices, cybercontrol, online jealousy, and online intrusive behaviors. Taking into account the objectives of our study, we selected the following subscales: cybercontrol, measured through 6 items (e.g., Item 1: “I have opened a fake account so that my partner can add me and I can control them”); online jealousy, measured through 4 items (e.g., Item 7: “I get jealous when my partner posts provocative photos on their social media profile”); and online intrusive behaviors, measured through 4 items (e.g., Item 11: “When I’m angry and my partner doesn’t answer, I leave them a lot of messages on their social network”). Responses are rated on scale a 5-point Lickert type ranging from 1 (never) to 5 (always). Higher scores indicate a higher level of cybercontrol, online jealousy, and online intrusive behaviors towards the partner. Cronbach’s alphas were 0.85 for Cybercontrol, 0.82 for Online Jealousy, and 0.84 for online intrusive behaviors in our study.

### 2.3. Procedure

The Institutional Review Board at the authors’ academic institution approved the study procedures. The participants for our study were recruited using the Computer-Assisted Web Interviewing (CAWI) system during September and October 2023. The procedure for using CAWI begins with defining the study’s objective to determine the necessary information and target audience. It employs specialized software to design visually appealing and easy-to-navigate surveys. CAWI allows for the real-time monitoring of responses and making necessary adjustments to ensure a high participation rate. CAWI is an online service where registered users complete tasks in return for a specified compensation. To receive compensation, participants had to complete the questionnaire; otherwise, they were eliminated from the study. Parents provided informed consent, and adolescents gave their assent. The adolescents completed the task using their parent’s CAWI account. A single online survey was accessed via an embedded link in the CAWI task. The survey assessed demographic characteristics (male, age, and sexual orientation) and the scales. The mean duration of the survey was 15 min.

### 2.4. Statistical Analysis

The following analyses were performed with IBM SPSS v.24 software. Firstly, we calculated the descriptive statistics and the mean differences as a function of gender in the variables and scales with Student’s *t*-test. Two age groups were established: younger adolescents aged 14 to 16 years (n = 159) and older adolescents aged 17 to 18 years (n = 157). Cohen’s d was also calculated. Second, we calculated bivariate correlations between the scales/subscales and the variables. Thirdly, we performed a three-step hierarchical linear regression, predicting the frequency of comments about women’s bodies. Finally, for each significant interaction among the study’s variables, we plotted the predicted values of the frequency of making comments about women’s bodies with high or low scores in partner cybercontrol, online jealousy, and hostile sexism.

## 3. Results

### Descriptive Statistics

Firstly, we compared the mean differences in the variables and scales of the study according to age. As can be seen in Table 1, there were significant differences in the variables (the frequency of comments, the effect of comments, and social expectations) and all the scales/subscales, with a variable effect size.

Younger boys (14 to 16 years old) made more comments about women’s bodies (t = 9.51, *p* < 0.001, d = 1.11) and had a higher positive perception of the impact of these comments on women’s bodies (t = 2.07, *p* < 0.039, d = 0.23). They also had higher social expectations about the practice of making comments about women’s bodies (t = 5.55, *p* < 0.001, d = 0.62) than older boys. However, both younger and older boys showed a high predisposition to stop making comments about women’s bodies if the women informed them of their discomfort, with nonsignificant differences (t = −0.937, *p* < 0.349, d = −0.10).

It can also be observed that younger boys obtained higher scores in the subscales of Hostile Sexism (t = 3.57, *p* < 0.001, d = 0.40), benevolent sexism (t = 5.22, *p* < 0.001, d = 0.59), partner cybercontrol (t = 11.7, *p* < 0.001, d = 1.53), online jealousy (t = 9.57, *p* < 0.001, d = 1.08), and online intrusive behaviors (t = 10.8, *p* < 0.001, d = 1.39) than older boys.

Table 2 presents correlation indices between the analyzed variables and subscales. The results show that the younger boys’ age, the greater the emission of comments about women’s bodies (r = −0.49, *p* < 0.01). They also have higher social expectations to make this type of comment (r = −0.29, *p* < 0.01), show a higher level of hostile sexism (r = −0.19, *p* < 0.01) and benevolent sexism (r = −0.28, *p* < 0.01), exert more partner cybercontrol (r = −0.57, *p* < 0.01), feel more online jealousy (r = −0.47, *p* < 0.01), and perform more online intrusive behaviors (r = −0.53, *p* < 0.01).

The more the number of comments about girls’ bodies (see Table 2), the greater the belief that women like to receive this type of comment (r = 0.25, *p* < 0.01). They think this type of comment has a positive effect on women (r = 0.25, *p* < 0.01); they have higher social expectations about making this type of comment (r = 0.60, *p* < 0.01). Also, boys who make more comments about women’s bodies show a higher level of hostile sexism (r = 0.29, *p* < 0.01) and benevolent sexism (r = 0.47, *p* < 0.01), exert greater partner cybercontrol (r = 0.73, *p* < 0.01), express higher online jealousy (r = 0.63, *p* < 0.01), and perform more online intrusive behaviors (r = 0.70, *p* < 0.01).

Next, the regression model was tested using hierarchical multiple regression to compare the strength of the prediction estimates of the variables (age, whether they think that women like to receive such comments, whether they think that such comments have a positive or negative effect on women, social expectations about making comments about women’s bodies, whether they would be willing to stop making such comments if they think they are harmful to women, ambivalent sexism, partner cybercontrol, online jealousy towards a partner, and online intrusive behaviors) in the frequency of making comments about women’s bodies (see Table 3). The five variables were entered at Step 1 of the analysis, accounting for a significant 49.5% of the variance.

In Step 2, the five predictors (hostile sexism, benevolent sexism, partner cybercontrol, online jealousy towards the partner, and online intrusive behaviors) were entered into the regression analysis, which accounted for a total of 64% of the variance in the model. When the predictor variables were added, they contributed an additional 14.5% to the variance, specifically in the frequency of emitting comment about women’s bodies, ΔR^2^ = 0.150, F(11, 305) = 51.9, *p* < 0.001. In the final model, Step 3, partner cybercontrol (β = 0.82, t = −2.67, *p* = 0.05) and online jealousy (β = 0.81, t = −2.29, *p* = 0.05) were significant.

All two-way interaction terms between variables and subscales were entered independently into Step 3 of the model using an interaction variable (Predictor × Predictor). These interactions explained an additional 9.7% (*p* < 0.05) of the variance of the frequency of comments about women’s bodies. However, only four of these interactions were statistically significant: age × partner cybercontrol (β = −0.72, t = −3.68, *p* = 0.01), hostile sexism × partner cybercontrol (β = 0.94, t = 1.95 *, *p* = 0.05), hostile sexism × online jealousy (β = −0.65, t = −2.09, *p* = 0.05), and benevolent sexism × social expectations (β = 0.57, t = 2.19, *p* = 0.05). All other combinations of interactions were nonsignificant.

**Figure 1:**
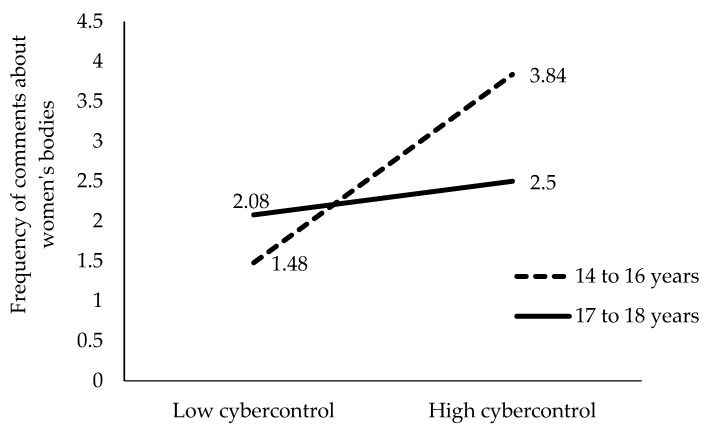
Moderating effect of age between partner cybercontrol and the frequency of comments about women’s bodies.

**Figure 2:**
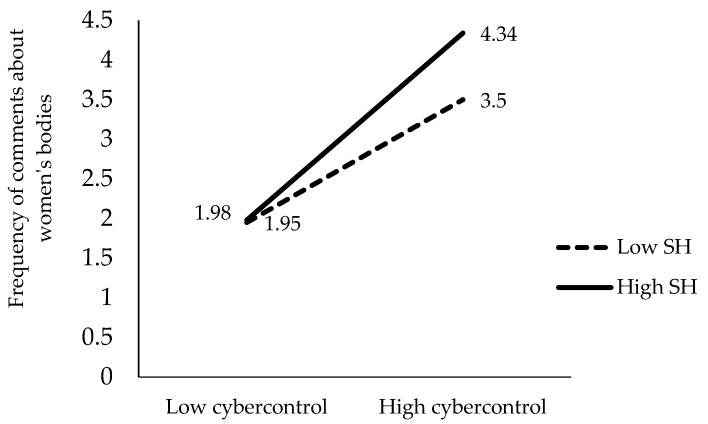
Moderating effect of hostile sexism (HS) between partner cybercontrol and the frequency of comments about women’s bodies.

**Figure 3:**
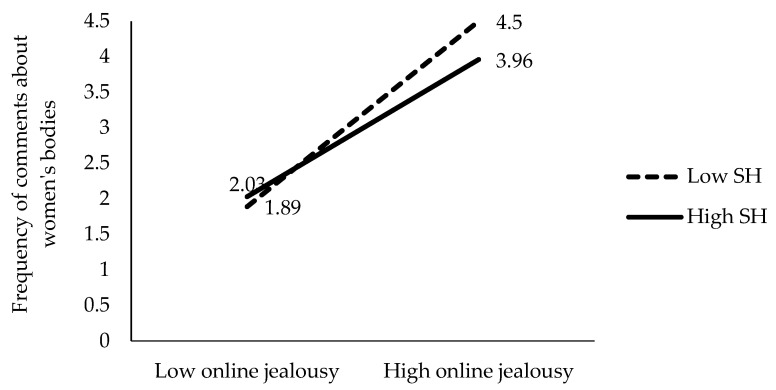
Moderating effect of hostile sexism (HS) between online jealousy and the frequency of comments about women’s bodies.

**Figure 4:**
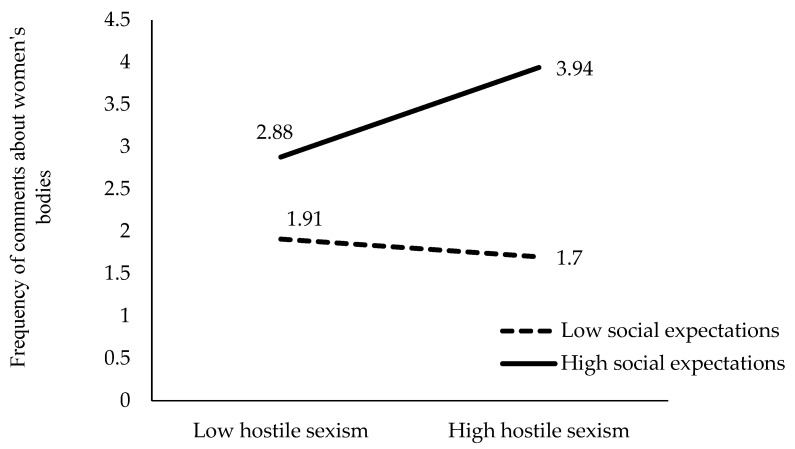
Moderating effect boys’ social expectations for making comments about women’s bodies between hostile sexism and the frequency of such comments.

To clarify the meaning of these four significant interactions of the hierarchical regression, we performed a detailed analysis of the mean scores of the frequency of comments about women’s bodies obtained by each group in each interaction. These mean scores for each group are represented in Figure 1, Figure 2, Figure 3 and Figure 4.

As shown in Figure 1, we compared the mean paired scores with a *t*-test. These comparisons indicate that levels of partner cybercontrol predict a greater emission of comments about women’s bodies both in younger boys aged 14 to 16 years (β = 0.17, t = 19.7, *p* < 0.01) and in older boys aged 17 to 18 years (β = 0.17, t = 2.37, *p* < 0.5).

The boys’ younger age and a higher level of partner cybercontrol predict a higher emission of comments about women’s bodies. Boys under 14 to 16 years with low partner cybercontrol are expected to make significantly more comments about women’s bodies than older 17 to 18 year olds with high partner cybercontrol (t = 4.85, *p* < 0.001) (Figure 1).

Similarly, we compared the mean scores using *t*-tests, as shown in Figure 2. The levels of partner cybercontrol positively predict the emission of comments about women’s bodies both at low (β = 0.47, t = 3.54, *p* < 0.001) and especially at high levels of hostile sexism (β = 0.84, t = 11.2, *p* < 0.001). Lower levels of hostile sexism predict fewer comments about women’s bodies both at a low level of partner cybercontrol (t = −0.151, *p* = 0.881) and particularly at a high level of partner cybercontrol (t = −0.2.58, *p* < 0.05).

As shown in Figure 3, we compared the mean paired scores using a *t*-test. These comparisons indicate that boys’ level of online jealousy better predicts comments about women’s bodies at both high (β = 0.70, t = 7.10, *p* < 0.01) and low levels of hostile sexism (β = 0.43, t = 3.19, *p* < 0.01). The highest emission of compliments is predicted by a high level of online jealousy, regardless of the level of hostile sexism.

As depicted in Figure 4, the relationship between boys’ level of benevolent sexism and their comments about women’s bodies is only positive and significant for boys’ high-level social expectations about making such comments (β = 0.51, t = 4.16, *p* < 0.001). For boys who show low social expectations for making such comments, the relationship between benevolent sexism and making such comments is negative, albeit nonsignificant (β = −0.12, t = −0.938, *p* = 0.353).

A higher level of boys’ social expectations for making comments about women’s bodies significantly predicts making more of these comments both in boys with a low level of benevolent sexism (t = −0.2.27, *p* < 0.001) and a high level of benevolent sexism (t = −5.02, *p* < 0.001).

## 4. Discussion

The purpose of the current study was to examine the frequency with which boys make verbal comments (compliments) to girls about their bodies, to know boys’ perceptions about the effect that these types of comments may have on girls, and discern whether they would be willing to stop making such comments if a girl or woman expressed their discomfort about them. We also assessed the level of ambivalent sexism and partner cyberviolence and confirmed their relationship with emitting “compliments” or comments about women’s bodies.

Concerning the frequency of compliments, adolescent boys have been observed to make this type of comment to girls, similar to studies with adult men (Oswald et al., 2019; Walton & Pedersen, 2022) and women, as women frequently report receiving comments about their bodies (Holland et al., 2017; Lameiras et al., 2018; Moya-Garófano et al., 2016, 2021). However, younger boys (14 to 16 years old), compared to the older ones, make more comments about women’s bodies and, at the same time, have a stronger perception of the positive impact of such comments. That is, they think that girls like to be complimented. In this sense, in the study of Walton and Pedersen (2022), 73.2% of the participants indicated that they expected the woman to feel flattered by the compliments. Boys and men misperceive women’s interest in receiving objectifying messages (Oswald et al., 2019), which leads to misinterpretations in their relationships with women, which, in turn, can lead to sexual violence towards women (Ward et al., 2015). However, an encouraging result obtained in this study for future interventions is that boys, regardless of age, would be willing to stop making comments about women’s bodies if women informed them of their discomfort.

Another interesting result in this study, in line with international and national studies, is that younger boys show a higher level of hostile and benevolent sexism (Gutiérrez et al., 2020; Walton & Pedersen, 2022) and carry out more cyberviolence behaviors towards their partners through cybercontrol, online jealousy, and online intrusive behaviors than older boys (Rodríguez-Castro et al., 2021). Our young people continue to reproduce gender stereotypes that are reflected in their sexist attitudes and frequently end up materializing in violence against their partner both offline and online, especially through cybercontrol (Rodríguez-Castro et al., 2021). Thus, when analyzing the relationship between the frequency of comments about women’s bodies, ambivalent sexism, and cyberviolence in relationships, we have found that boys who make more comments about women’s bodies show a higher level of hostile sexism and benevolent sexism (Harsey & Zurbriggen, 2021; Walton & Pedersen, 2022; Wright & Tokunaga, 2016), exert greater partner cybercontrol, and show greater online jealousy and online intrusive behaviors.

Finally, our focus was on determining which study variables predict the frequency of comments about women’s bodies and on verifying the moderating role of these variables in a group of adolescent boys. This is the first study that examines the combination of these variables in a large sample. Our results have identified the following predictors of boys’ frequency of comments about women’s bodies: age; predisposition to change; partner cybercontrol; online jealousy; and four interactions between the variables combining the effect of age and partner cybercontrol, hostile sexism and partner cybercontrol, hostile sexism and online jealousy, and benevolent sexism and social expectations about making comments about women’s bodies. Age, hostile sexism, and benevolent sexism modulate the frequency of comments about women’s bodies.

Subsequent analyses yielded four relevant results. Firstly, boys who engage in partner cybercontrol behaviors, regardless of age, are likely to make the most comments about women’s bodies. But younger boys (14 to 16 years old) and those who exert the most partner cybercontrol are predicted to make the most comments about women’s bodies. This result is very interesting to include in intimate partner violence prevention programs, both online and offline (Rodríguez-Castro et al., 2022).

Secondly, the level of partner cybercontrol shown by boys predicts making comments about women’s bodies in boys with a low level of hostile sexism and especially in boys with a high level of hostile sexism. That is, it is predicted that the least hostile sexist boys, when compared to the more hostile sexists, will make fewer comments about women’s bodies at both low and high levels of cyberviolence. In this sense, the study by Walton and Pedersen (2022) reaffirms that boys and men who “compliment” girls and women show a higher level of hostile sexism and greater tolerance towards sexual harassment.

Thirdly, boys who make comments about women’s bodies show a high level of online jealousy, regardless of their level of hostile sexism. Finally, the higher the social expectations boys have about making comments about women’s bodies, the more comments they will make regardless of their level of benevolent sexism. Younger boys believe that society expects them to make these kinds of comments to girls. As shown in various studies (Rodríguez-Castro et al., 2018; Setty, 2019; Ward et al., 2015), boys make sexual comments about girls’ bodies due to social pressure from the peer group, to be socially accepted by their peers, and to increase their popularity and/or their “masculinity” value.

These results encourage going one step further and reflecting on why male and female adolescents make comments about girls’ bodies. In the pre-adolescent and adolescent stage, which is characterized by the construction of identity and the beginning of the first socio-affective and sexual relationships, adolescents are educated through the process of differential socialization based on gender stereotypes, assuming that power and sexual dominance are associated with masculinity (Bareket & Shnabel, 2020), which translates into behaviors of the sexual objectification of girls and women (Rousseau & Eggermont, 2017; Tiggemann & Slater, 2015). Thus, boys adopt a position of power towards girls, treating them as instruments to satisfy their masculine needs and pleasure (MacKinnon, 1987), with sexual objectification as a form of oppression of girls and women. This dynamic of issuing compliment is normalized in homosocial contexts, where boys are more likely to justify gender stereotypes, show sexist attitudes, accept rape myths, and perpetuate sexual harassment and/or abuse behaviors (Moya-Garófano et al., 2021, 2022). At the same time, adolescent girls learn to assume a “submissive” and “passive” role, wherein their worth is primarily defined by their physical appearance (Lameiras et al., 2018), which means that they are represented and judged solely by the sexual parts or functions of their body, ignoring their personality and subjectivity.

### Limitations and Future Directions

This study makes some important contributions; however, some limitations should be mentioned. Our participants were recruited online, and this could create some bias. Although using studies based on online surveys has become prevalent, this strategy can also lead to selection bias and data invalidity (Kennedy et al., 2020).

Finally, although compliments are used in several countries, all boy participants in our study were from Spain, and so we cannot generalize the findings to other countries; thus, conducting studies in different countries where compliments are also frequent could enrich our understanding of this phenomenon.

## 5. Conclusions

This study has shown how the objectification of women’s bodies through compliments, cyberviolence in relationships, and the level of hostile sexism are part of the socio-affective relationships of adolescents. It is essential to encourage adolescents’ critical thinking about the negative consequences of sexual objectification behaviors of the bodies of girls and women to eradicate these sexist and violent practices in interpersonal relationships. It is also important to focus on the more “subtle” forms of sexual objectification that can be interpreted as a “flattering” or “enjoyable” comment for girls and women, especially if the comment is made by a partner, as it is more difficult to identify the negative consequences and to combat sexual violence behaviors.

Therefore, as a measure to prevent sexual harassment through comments on women’s bodies, gender-based cyberviolence, and sexism, we consider it important for the educational system to promote comprehensive sex education to eradicate the rigid model of gender stereotypes and move towards a co-educational model based on “equality” in which boys and girls learn to build relationships free from violence, both online and offline.

## Figures and Tables

**Table 1:** Differences in means of scales/subscales and effect size by age.

	Mean (SD)		t	*p*	Cohen’s d	Effect Size R
	14–16 Years	17–18 Years				
Frequency of comments about women’s bodies	3.12 (1.07)	2.16 (0.592)	9.51	0.001	1.11	0.48
You think women like to receive feedback about their body	3.78 (1.03)	3.73 (0.990)	0.409	0.683	0.04	0.02
Effect on girls of comments about the body	3.41 (0.649)	3.21 (1.05)	2.07	0.039	0.23	0.11
Social expectations of making comments about the body	3.78 (1.06)	3.14 (1.00)	5.55	0.001	0.62	0.29
Predisposition to change	5.35 (0.768)	5.45 (1.14)	−0.937	0.349	−0.10	0.05
Hostile sexism	3.96 (1.00)	3.51 (1.16)	3.57	0.001	0.40	0.19
Benevolent sexism	3.76 (1.12)	3.09 (1.12)	5.22	0.001	0.59	0.28
Partner cybercontrol	3.14 (1.17)	1.76 (0.815)	11.77	0.001	1.53	0.61
Online jealousy	3.31 (1.08)	2.21 (0.949)	9.57	0.001	1.08	0.47
Online intrusive behaviors	3.13 (1.16)	1.85 (0.853)	10.86	0.001	1.39	0.57

**Table 2:** Pearson correlations between the various scales/subscales.

	Age	1	2	3	4	5	6	7	9
(1) Frequency of comments about women’s bodies	−0.49 **								
(2) You think women like to receive comments about their body	0.11	0.25 **							
(3) Effect on girls of comments about the body	−0.11	0.25 **	0.26 **						
(4) Social expectations about making comments about the body	−0.29 **	0.60 **	0.28 **	0.28 **					
(5) Predisposition to change	−0.05	−0.07	−0.10 **	−0.10	−0.03				
(6) Hostile sexism	−0.19 **	0.29 **	0.18 **	0.18 **	0.38 **	0.05			
(7) Benevolent sexism	−0.28 **	0.47 **	0.21 **	0.23 **	0.48 **	0.01	0.57 **		
(8) Partner cybercontrol	−0.57 **	0.73 **	0.23 **	0.23 **	0.49 **	−0.18 **	0.24 **	0.46 **	
(9) Online jealousy	−0.47 **	0.63 **	0.20 **	0.21 **	0.48 **	−0.06	0.32 **	0.49 **	0.80 **
(10) Online intrusive behaviors	−0.53 **	0.70 **	0.22 **	0.22 **	0.51 **	−0.17 **	0.25 **	0.49 **	0.89 **

Note: ** *p* < 0.01.

**Table 3:** Hierarchical multiple regression analysis predicting the frequency of making comments about women’s bodies.

	*B*	*SE B*	β	*t* (*p*)
**Step 1**				
Age	−0.18	0.08	−0.362	−8.56 ***
Social expectations about making comments about the body	0.20	0.04	0.408	8.66 ***
You think women like to receive comments about their body	0.14	0.04	0.174	3.76 ***
Effect on girls of comments about the body	0.02	0.03	0.043	1.01
Predisposition to change	0.01	0.03	−0.076	−1.82
*F* (*df*, *df* _error_)			62.6 *** (5, 310)	
*R* ^2^			0.495	
**Step 2**				
Age			−0.095	−2.16 *
Social expectations about making comments about the body			0.230	5.20 ***
You think women like to receive comments about their body			0.154	3.66 ***
Effect on girls of comments about the body			0.022	0.598
Predisposition to change			0.008	0.212
Hostile sexism	−0.03	0.03	−0.042	−0.953
Benevolent sexism	0.02	0.03	0.029	0.610
Partner cybercontrol	0.30	0.06	0.385	4.57 **
Online jealousy	−0.01	0.05	−0.022	−0.355
Online intrusive behaviors	0.10	0.06	0.126	1.58
*F* (*df*, *df* _error_)			51.9 *** (11, 305)	
*R* ^2^			0.640	
Δ*R*^2^			0.150	
ΔF^2^			21.9 ***	
**Step 3**				
Age	1.48	0.36	0.768	4.09 ***
Social expectations about making comments about the body	−0.01	0.11	−0.019	−0.148
You think women like to receive comments about their body	−0.07	0.12	−0.073	−0.578
Effect on girls of comments about the body	0.22	0.15	0.209	1.46
Predisposition to change	−0.21	0.10	−0.223	−2.12 *
Hostile sexism	−0.46	0.28	−0.536	−1.63
Benevolent sexism	0.19	0.30	0.233	0.629
Partner cybercontrol	0.65	0.35	0.820	−2.12 *
Online jealousy	0.68	0.30	0.817	2.29 *
Online intrusive behaviors	−0.17	0.38	−0.219	−0.459
Age × partner cybercontrol	−0.48	0.13	−0.729	−3.68 ***
Hostile sexism × partner cybercontrol	0.15	0.07	0.945	1.95 *
Hostile sexism × online jealousy	−0.10	0.05	−0.657	−2.09 *
Benevolent sexism × social expectations	0.08	0.03	0.571	2.19 *
*F* (*df*, *df* _error_)			28.5 *** (32, 283)	
*R* ^2^			0.737	
*R*^2^			0.111	
ΔF^2^			6.31 ***	

Note. * *p* < 0.05. ** *p* < 0.01. *** *p* < 0.001.

## Data Availability

The data presented in this study are available from the corresponding author on reasonable request. Code availability (software application or custom code): The original contributions presented in this study are included in the article. Further inquiries can be directed to the corresponding author(s).
